# Review: Research Toward Safer Resection of the Cirrhotic Liver

**DOI:** 10.1155/2000/31945

**Published:** 2000-01

**Authors:** M. A. J. Moser, N. M. Kneteman, G. Y. Minuk

**Affiliations:** 1 Liver Diseases Unit Departments of Surgery, Medicine, and Pharmacology University of Manitoba Winnipeg Manitoba Canada; 2 Department of Surgery University of Alberta Edmonton Alberta Canada; 3 Liver Diseases Unit Health Sciences Center 820 Sherbrook Street Winnipeg Manitoba R3A 1R9 Canada

## Abstract

Despite recent advances in hepatic surgery, resection of the cirrhotic liver continues to be fraught with high morbidity and mortality rates. As a result, for many patients requiring resection of HCC the postoperative course is complicated and the probability of cure is diminished by coexisting cirrhosis. In this review, we discuss the characteristics of the cirrhotic liver which make it poorly tolerant of resection and the most common complications that follow such surgery. The main purpose of this paper is to review recent attempts to identify interventions that might be beneficial to cirrhotic patients undergoing resection. These interventions include assessment of liver reserve, advances in surgical technique, and improvement in liver function and regeneration.


*HPB Surgery*, 2000, Vol. 11, pp. 285–297
Reprints available directly from the publisher
Photocopying permitted by license only

© 2000 OPA (Overseas Publishers Association) N.V.
Published by license under
the Harwood Academic Publishers imprint,
part of The Gordon and Breach Publishing Group.
Printed in Malaysia.

### Review: Research Toward Safer Resection of the Cirrhotic Liver

M. A. J. MOSER[a,b], N. M. KNETEMAN[b] and G. Y. MINUK[a,*]

[a]*Liver Diseases Unit, Departments of Surgery, Medicine, and Pharmacology, University of Manitoba, Winnipeg, Manitoba, Canada;* [b]*Department of Surgery, University of Alberta, Edmonton, Alberta, Canada*

*(Received 10 November 1998)*

**Despite recent advances in hepatic surgery, resection of the cirrhotic liver continues to be fraught with high morbidity and mortality rates. As a result, for many patients requiring resection of HCC the postoperative course is complicated and the probability of cure is diminished by coexisting cirrhosis. In this review, we discuss the characteristics of the cirrhotic liver which make it poorly tolerant of resection and the most common complications that follow such surgery. The main purpose of this paper is to review recent attempts to identify interventions that might be beneficial to cirrhotic patients undergoing resection. These interventions include assessment of liver reserve, advances in surgical technique, and improvement in liver function and regeneration.**

*Keywords:* Cirrhosis, resection, regeneration, liver function, morbidity and mortality

### INTRODUCTION

In the last 20 years, surgeons appear to have become more aggressive with hepatic resections and as a result, the number of cases has been steadily increasing. Tumors which were once considered unresectable are now being approached surgically, particularly in the case of colorectal metastases. Hepatic resection is possible in large part because of the tremendous regenerative ability of the normal liver. In a patient with a normal liver, up to 75% of the liver mass can be safely resected, and mortality rates of approximately 5% or less are possible [1].

Resection of the cirrhotic liver, however, presents a difficult problem and until recently, cirrhosis was considered a contraindication for hepatic resection. Mortality rates of 20–50% are commonly quoted, despite careful preoperative workups and rejection of large numbers of patients who are medically unfit or have lesions that are considered unresectable [2, 3].

The most common indication worldwide for hepatic resection is hepatocellular carcinoma (HCC), which constitutes approximately 90% of all malignant primary liver tumors. Depending on the geographic location, 50–85% of patients with HCC have associated cirrhosis [4, 5]. Conversely, 10–20% of patients with cirrhosis from any etiology, will go on to develop HCC [3, 6].

*Address for correspondence: Liver Diseases Unit, Health Sciences Center, 820 Sherbrook Street, Winnipeg, Manitoba, R3A 1R9. Tel.: (204) 787-4662, Fax: (204) 775-4255.

Thus, the majority of patients who require resection also have cirrhosis or impaired liver function. An increased ability to safely perform resection in these patients should also lead to an increase in the surgical cure rate of hepatic malignancies, both primary and metastatic.

### THE CIRRHOTIC LIVER

There are a number of factors that make it difficult for a cirrhotic patient to tolerate resection, including poor functional reserve, impaired regeneration, elevated portal venous pressures, and the characteristics of the patient population undergoing resection.

### Reduced Function

The removal of functional liver tissue from an organ that already has marginal function can lead to many complications. If insufficient functioning hepatic parenchyma remains after resection, the patient will likely develop liver failure, which is the leading cause of death postoperatively. It is crucial to be able to resect a sufficient amount of liver to effect a cure, while leaving enough healthy liver to ensure survival. A minimum amount of liver function appears to be crucial to sustain life.

### Impaired Regeneration

Unlike the *normal* liver, which, within 3–12 months can regenerate to its original size and function, even after 80% resection, the cirrhotic liver has impaired regenerative ability. Indeed, until recently, the cirrhotic liver was thought to be unable to regenerate [7]. However, we now know that while it is able to regenerate somewhat, the cirrhotic liver is greatly impaired both in *rate* and *extent* of regeneration [8]. The reason for this impaired regenerative capacity is currently the subject of numerous investigations, but increased levels of circulating growth inhibitors such as ammonia, mercaptans, and GABA, rather than a paucity of growth stimulators is thought to be operative [9, 10].

### Portal Hypertension

Portal hypertension poses a number of problems in cirrhosis. Principal amongst these is that the rate of blood loss is accelerated and hemostatic mechanisms are often unable to overcome venous bleeding because of the high pressures. In addition, numerous venous collaterals are formed in an attempt to bypass the high resistance of the intrahepatic vasculature. These collaterals are particularly prominent in the area of the porta hepatis, making dissection difficult and hazardous. This likely contributes to the high mortality rate noted in the performance of cholecystectomy in cirrhotic patients [11, 12]. Kanematsu noted an immediate increase in portal pressures at the time of major hepatic resection. Smaller (segmental and subsegmental) resections, on the other hand, did not lead to significant increases in pressure. Morbidity and mortality did not, however, correlate with the degree of increase in portal pressure [13].

### Patient Population

The majority of patients requiring hepatic resection (particularly alcoholics with HCC), are in their 5th to 7th decades of life and will often have associated medical problems. Moreover, there is evidence to suggest that a number of hepatic changes are seen with aging, including decreased Reticuloendothelial System (RES) function [14], decreased regeneration, and impaired mitochondrial enzyme activity [15]. Careful preoperative assessments and vigilant post-op monitoring are essential in managing these patients.

### RISKS AND COMPLICATIONS OF LIVER RESECTION IN CIRRHOSIS

Hepatic resection in non-cirrhotic patients can be accomplished with relative safety, and a morbidity of under 5% for elective lobar resections is commonly achievable in most centers. The main risk factor appears to be associated medical illness [16].

On the other hand, the risks and complications associated with resections in cirrhotic patients are more complex. The extent of resection and degree of baseline functional impairment appear to be the main independent risk factors. Other factors, such as bleeding tendency, ascites, anemia, and hypoalbuminemia appear to be dependent on these two variables [17, 18]. The serious complications that these patients are prone to develop are hepatic failure, sepsis, and bleeding, which can largely be attributed to insufficient hepatic function.

Cirrhotic patients are prone to these complications when undergoing surgery of any kind, not only hepatectomy. Doberneck examined cirrhotic patients undergoing a variety of operations including intraabdominal, genitourinary, and orthopedic procedures [19]. An overall complication rate of 47.1% along with a mortality rate of 19.6% was seen. Liver failure occurred in 43 patients, sepsis in 19 patients, and bleeding in 9 out of 102 patients.

In 1985, Yanaga noted intraperitoneal septic complications in 16 of 103 cirrhotic patients undergoing hepatic resection [20]. On the other hand, only 3 of 46 patients without cirrhosis developed sepsis. The cause of death in the 13 patients that died was noted as progressive liver failure.

Another large series identified a mortality rate of 8.9% and a complication rate of 31.7% among 101 cirrhotic patients undergoing major and subsegmental resections for HCC [21]. Sepsis was seen in 4 of 102 patients, bleeding in 10 patients, and liver failure in 10 patients. The lower mortality rate seen here can be explained by the fact that over half the resections were subsegmental and a large number were done for palliation only.

More recently, Fan in 1995 published results of a series of 54 cirrhotic patients undergoing major resections [22]. Seven deaths occurred for a mortality of 13%, and serious complications were seen in 50%. Sepsis complicated the early postoperative course in 18 patients, while excessive bleeding was seen in 4 patients. No mention was made of the incidence of liver failure.

### Hepatic Failure

The term "hepatic failure" does not have a precise definition and as a result, the reported incidence of this complication varies widely. Liver failure is, however, consistently quoted as the most common cause of death in cirrhotic patients early in the postoperative period. One author has proposed that hepatic failure includes progressive jaundice, ascites, and encephalopathy, in addition to rapidly escalating liver function studies [14]. Liver failure can also result in insufficient metabolic, synthetic, and immune function, predisposing to the other complications of encephalopathy, jaundice, hypoalbuminemia, hypoglycemia, and sepsis.

If after estimating post-operative liver function, it appears that insufficient liver function will remain, it is essential to reduce major resections, by considering segmentectomy or even subsegmental resection. The most precise means of estimating post-operative liver function is discussed later in this review.

### Sepsis and ARDS

Cirrhotic patients undergoing resections are predisposed to infection, both locally and systemically. This is likely related to the loss of RES function due to resection [14].

In Yanaga's study of 149 patients undergoing resection, most of whom were cirrhotic, perioperative variables associated with Intraperitoneal Septic Complications after Hepatectomy (IPSCH) [20] were examined. Risk factors identified included right lobectomy, age greater than 65 years, operation time greater than 5 hours, blood loss >3000 mL, and postoperative bleeding requiring reoperation. Notably, the risk of IPSCH did not correlate with preoperative liver function tests nor with the presence of cirrhosis. The authors postulated that this relates to the fact that more limited resections were carried out in cirrhotic patients.

The liver's role in filtering blood prior to entry into the lungs may in part explain the increased risk of ARDS following surgical resection of cirrhotic livers [14].

### Bleeding

The factors predisposing the cirrhotic patient to bleeding are multiple and have been studied extensively. First of all, decreased hepatic synthetic function leads to low levels of clotting factors. The result is an abnormal PT/PTT. Due to the short half life of clotting factors (most under 1 day), PT is a sensitive indicator of acute changes in liver synthetic function [11].

In addition, thrombocytopenia, associated with splenomegally, is often seen with chronic liver disease. Nonetheless, platelet counts rarely drop to low enough levels to contribute primarily to bleeding.

Portal Hypertension is also a factor in increased bleeding and collateral neovascularisation makes dissection difficult, particularly in the area of the porta hepatis. The classic paper by Schwartz in 1981 describes massive bleeding in 18 of 33 patients who underwent cholecystectomy or an operation for bile duct obstruction, despite the presence of normal coagulation profiles in approximately half the patients [12]. On the other hand, among patients undergoing cholecystectomy at the same time as a portal decompressive procedure, significant bleeding occurred in only one of 7 patients [12]. These findings emphasize the role elevated portal pressures play in contributing to bleeding associated with biliary tract surgery. A number of studies have examined the role of intraoperative Vasopressin as a means of decreasing portal pressure and thereby limiting perioperative bleeding problems. During shunt operations, Sirinek *et al.*, reported a 28% decrease in portal pressure with Vasopressin, which was associated with a decrease in blood loss, and a shorter operating time [23].

Over the last 20 years, the existence of a fibrinolytic state as well as the extent of its contribution to the problem of operative and postoperative blood loss has been debated. When properly diagnosed, bleeding associated with a fibrinolytic state has been successfully controlled with the careful use of aminocaproic acid [12]. Another antifibrinolytic is Aprotinin (Trasylol®), a serine protease inhibitor which inhibits plasmin and kallikrein. It has been used successfully as an antifibrinolytic agent in cardiac surgery and liver transplantation [24], and may similarly be beneficial to cirrhotic patients undergoing resection.

It is crucial to correct hemostasis preoperatively and to monitor hemostatic function closely postoperatively. Recent advancements in critical care have contributed significantly to the decrease in bleeding complications [25].

### POSSIBLE INTERVENTIONS

There are a number of approaches that can be undertaken to improve the outcome of cirrhotic patients undergoing hepatic resection:

1. Optimization of liver function and structure preoperatively.
2. Careful preoperative estimation of the extent of resection possible to allow for adequate postoperative hepatic function.
3. Advancements in surgical techniques.
4. Enhancement of post-operative hepatic regeneration.

5. Perioperative monitoring and management of nutritional, clotting, septic, and fluid balance problems.

### ASSESSMENT OF HEPATIC RESERVE

A reliable estimate of liver function is essential in planning resection in patients with impaired hepatic function. Most importantly, a method of estimating post-operative function could suggest how much resection is feasible, if any. Many systems and tests have been devised to evaluate liver function; many are impractical in a clinical setting, and few are in common use today.

### Serum Liver Panel

Aside from one report suggesting that elevated serum bilirubin preoperatively correlates with poor outcome from resection [26], there is agreement that bilirubin, Aspartate Aminotransferase, and alkaline phosphatase levels preoperatively do not provide a reliable estimate of hepatic reserve and are not useful in gauging prognosis after liver resection [27].

### Indocyanine Green (ICG) Retention and Clearance

ICG is a safe, nontoxic dye that is bound to albumin and is cleared almost exclusively by the liver. The rate at which ICG is cleared from the blood after intravenous injection is a measure of hepatic blood flow. ICG retention after 15 minutes, a variation of the test, can be done at the bedside. Both tests have been shown by several authors to be sensitive measures of liver impairment, and have been used in conjunction with other tests to better assess liver resection and estimate post resectional function [28].

In Fan and Lai's multivariate analysis of 54 cirrhotic patients, preoperative ICG clearance was the only useful preoperative measure in predicting postoperative liver failure. Discriminant analysis further revealed that an ICG clearance of 14% most reliably separated survivors and non-survivors [22]. In a similar way, $ICG_{15}$ was one of the major factors in Yamanaga's regression equation [17], which will be discussed later.

Recently, Scudamore looked at preoperative tests in a similar fashion on 22 cirrhotic patients undergoing resection [29]. Again, ICG clearance was the only significant factor in predicting postoperative liver failure. Using discriminant analysis, the critical ICG clearance was found to be 5 ml/min/kg. It was suggested that patients whose ICG clearance is lower than the critical level should not undergo hepatic resection.

Overall, ICG clearance and ICG retention are relatively simple and safe tests, and appear to be the most reliable assessments of liver functional reserve currently available.

### Cytochrome A ( $+A_3$) Assay of Mitochondria

This test is based on the fact that hepatocytes require enhancement of phosphorylative capacity of mitochondria in order to undergo regeneration. When respiratory carriers such as $+a_3$ are reduced, this enhancement cannot occur and regenerating processes are impaired. Patients with reduced cytochrome a show a high rate of complications (80%) and mortality (40%) associated with formal lobectomy. It has been suggested that these patients should have the extent of resection reduced or the operation avoided if possible. A disadvantage to the test is that $+a_3$ levels must be obtained from a liver biopsy specimen [15]. Since the initial report in 1973, little has been done to pursue this test as a means of assessing hepatic reserve preoperatively.

### $^{198}$Au- Colloid Clearance

Another means of assessing liver function based on blood flow is the determination of the clearance of gold colloid by the liver. This technique is based on the rate of labeled colloid uptake by Kupffer cells as measured by a gamma camera in many small individual sectors. Using computerized techniques, a blood flow index ($KL_{Au}$) can be calculated and it is possible to estimate with considerable accuracy what $KL_{Au}$ will be when certain "sectors" of liver tissue are removed. An estimated post op $KL_{Au}$ of less than 50 is predictive of a poor outcome and increased mortality [30].

### Combination of Measures

Based on the concept that a certain quantity and quality of liver tissue is required to sustain life, Yamanaka and colleagues have developed a combination of measures to preoperatively predict the volume and function of the liver following partial hepatectomy.

In their hands, *quantity* of remaining liver was estimated from CT scan, including volume of parenchyma to be removed and volume remaining, while excluding volume of tumor, which constitutes non-functioning tissue. These estimates correlated strongly with actual volumes of the surgical specimens. Finally, Parenchymal Hepatic Resection Rate (PHRR) is calculated to more accurately represent the percentage of *functioning* hepatic parenchyma that will be removed:

$$\frac{(\text{Volume of liver to be resected} - \text{volume of tumor})}{(\text{Total liver volume} - \text{volume of tumor})}$$

The *quality* of liver or liver function is assessed by ICG retention after 15 minutes.

When the 38 patients in their study were classified based on PHRR and $ICG_{15}$, a linear separation was seen between survivors and non-survivors [31].

A year later, the same group formulated a multiple regression equation for prediction of postoperative liver failure [5]. They examined 17 variables and found the strongest associations with PHRR, $ICG_{15}$, and patient age, which lent support to their concept of 'quantity and quality'. The regression equation was:

$$PS = -84.6 + 0.93PHRR + 1.11\ ICG_{15} + AGE$$

The equation provides a 'prognosis score' (PS); scores greater than 50 are highly predictive of death from liver failure.

This same group has recently published a prospective study describing 10 years experience using this equation on 434 patients undergoing liver resection, most of whom had cirrhosis [13]. Patients with scores greater than 55 were classified as high risk, whereas scores of 45–55 were considered borderline. The objective was to obtain a score of 45 or less (the low risk group) by changing the resection from a lobectomy to a lesser resection whenever possible. For patients with scores greater than 50, medical management was strongly advised.

Overall, 129 patients had the extent of their resections reduced preoperatively. Among the seven patients who had scores higher than 55, six died within 3 months post op, all due to liver failure. Among patients with scores under 45, a mortality of 7.3% was achieved (26/357) for HCC, while none of 49 patients with metastatic cancer died. The group of patients with PS of 45 to 55 were identified as borderline; in this group, a striking difference in Oral Glucose Tolerance Test (OGTT) results was noted between survivors and non-survivors. On this basis, the authors suggest placing patients with borderline scores and linear OGTT curves into the high-risk category as a refinement of their assessment technique. Resection may be considered relatively safe for patients in the borderline zone who have parabolic OGTT curves.

### SURGICAL CONSIDERATIONS

The development of subsegmental resection (SSR) techniques and the use of intraoperative ultrasound (IOUS) have been significant advancements in liver surgery. Subsegmental resection is particularly helpful in resection of cirrhotic liver to minimize the extent of resection of non-diseased liver. Using IOUS, the tumor is identified as well as its relation to surrounding vessels. A needle is placed in the branch of the portal vein supplying the tumor and ICG or indigo carmine dye is injected to demarcate the area to be resected. Dissection along the demarcation line allows for the resection to be carried out with minimal bleeding [32]. IOUS is helpful in assessing the feasibility of resection as well as obtaining an accurate assessment of the extent of disease [32]. Relationships of the tumor to vascular structures as delineated by IOUS influenced the choice of operative procedure in 22 of 45 patients (49%) in one study [33] and 20 of 30 patients (67%) in another [34]. In the case of surgery for metastatic disease, IOUS was more sensitive than CT or preoperative ultrasound in detecting occult metastases under 1 cm in size [33].

Other interventions have also been proposed, particularly methods to decrease the bleeding complications associated with cirrhosis. Techniques of occluding hepatic inflow (Pringle maneuver) and outflow are being investigated as means of reducing blood loss in healthy livers [16]. However, the ability of cirrhotic liver to tolerate blood flow occlusion has not yet been fully elucidated and may limit its use in this situation [35]. In a pilot study involving 15 patients with cirrhosis who had hepatic inflow occlusion (Pringle maneuver) for 9–32 minutes during hepatic resection, a significant decrease in blood loss was achieved compared to a similar group of 15 patients operated on without the use of blood flow occlusion. In addition, no hemodynamic complications resulted and no changes were seen in liver function studies, followed for up to 3 weeks post op in the blood flow occluded group [36]. Overall, the cirrhotic liver appeared to tolerate ischemia better than originally thought, possibly due in large part to hepatic venous backflow. A later study looked at 44 cirrhotic patients and the use of inflow occlusion for 8 to 46 minutes without adverse effects, whereas simultaneous inflow and outflow occlusion, while it resulted in less blood loss, was associated with liver enzyme changes consistent with ischemia. At the end of the study, the need for a future prospective study was emphasized [37].

A number of techniques have been developed to aid in the hemostatic dissection of the liver, including the Cavitron ultrasonic aspirator (CUSA), the Argon beam, and YAG laser. Few of these have been subjected to prospective studies, particularly in the setting of cirrhosis. The CUSA may be impractical in cirrhotic liver, because the parenchyma is of a rubbery texture, closely resembling that of the major blood vessels. On the other hand, a water-jet dissector could be useful to decrease blood loss in cirrhotic patients [38, 39].

The use of the Lin liver clamp [40] is controversial, particularly in cirrhosis, because of the inhomogeneous texture and fibrous scarring present [16]. In addition, the neovascularisation associated with portal hypertension makes its application difficult and hazardous at times. A transdiaphragmatic approach to the right lobe has been advocated by one group to avoid the hypervascular porta hepatis area [41]. Using this approach, a decrease in blood loss and post op complications was achieved. On the other hand, the choice of a thoracic incision needs to be made carefully, because it too has a high rate of complications; also, hepatic inflow occlusion and vascular control are not possible from this incision.

### POSSIBLE FUTURE INTERVENTIONS

### Improvement of Liver Function Preoperatively

In cirrhosis, repair and regeneration in response to an insult result in fibrosis and architectural distortion of both parenchymal and vascular elements [42]. The precise relationship between fibrosis and liver function or regenerative potential requires further investigation [43]. It is well known that the advanced cirrhotic liver possesses poor regenerative ability. As a result, a number of studies have attempted to reduce or prevent fibrosis and improve regeneration [44].

*Colchicine* has been studied for many years and its effects have been well documented. This agent acts on many phases of collagen metabolism including decreased production and secretion, while increasing collagenase production [45]. In addition, colchicine affects lymphocyte and monocyte activity and these cells may contribute to the stimulation of myofibroblasts in the liver. The actions of colchicine appear to be on the incorporation of proline and independent of impairment of prolyl hydroxylase activity [44]. *In vivo* studies in the rat have suggested decreased fibrinogenesis and improved liver function [46].

A randomized controlled trial of Colchicine followed 100 patients for 3 to 14 years [47]. Histological improvement was suggested in the group receiving colchicine. Survival was also improved. Five year survival rates were 75% in the colchicine group *versus* 34% in the placebo group, while ten-year survival rates were 56% and 20% respectively. Long-term colchicine improved patient survival in cirrhosis, by preventing and reversing fibrosis. However, the colchicine and placebo treated groups were not well matched, and drop out rates were high. Moreover, it is not clear whether the beneficial effects can occur rapidly enough to be practical for preoperative preparation.

*Pentoxifylline* is a methylxanthine which has been used for years to improve blood flow in the lower limbs of patients with intermittent claudication. In a number of studies involving animal models of cirrhosis, pentoxifylline has been shown to decrease hepatic fibrinogenesis and improve serum liver function tests [48, 49]. The mechanism involved is that of inhibition of Platelet-Derived Growth Factor (PDGF) induced fibrinogenesis, possibly by interfering with post-receptor second messenger signals involving the adenosine receptor. Other studies have shown a protective effect of adenosine in a rat model of cirrhosis [50].

*Ciprofloxacin,* in addition to its antibiotic actions, has intrinsic GABA receptor blocking properties [9]. GABA receptors and transport proteins have been demonstrated on the surface of hepatocytes [51] and it has been shown that GABA has an inhibitory effect on hepatic regeneration [52]. In addition, elevated GABA levels are a common finding in the setting of acute or chronic liver disease [53]. Studies have shown that ciprofloxacin through its GABA blocking properties, enhances regeneration and protects against ethanol- and galactosamine-induced inhibition of regeneration [54]. This effect was independent of the antibiotic effect on the intestinal flora [55].

A recent study has demonstrated a reduction in the degree of fibrotic change in cirrhosis, after a 4 week course of Ciprofloxacin in the $CCl_4$ induced cirrhotic rat model. In addition, liver function as measured by serum ALT was improved [56]. The effects on regeneration, fibrosis, and hepatocyte viability may be beneficial to the cirrhotic liver undergoing partial hepatectomy. Such studies are currently underway.

*Traditional Chinese Herbal Remedies* have been used for centuries in the treatment of liver disease. While many formulations exist, few have been well studied in a controlled scientific fashion. One specific formulation which contains many ingredients, including boiled turtle shell, leech, pangolin scales, and ginseng was found in a prospective, controlled trial to have a beneficial effect on the extent of hepatic fibrosis in a rat model of cirrhosis [56]. Fibrosis and architectural distortion appeared to be reversed almost to normal. The mechanism of action involved and which of the components is active remains to be elucidated.

*Hepatocyte Growth Factor (HGF)* was first discovered in 1982 and is now considered the most potent stimulator of DNA synthesis in hepatocytes [57]. The many effects of HGF *in vitro* have been well documented; these include increased DNA synthesis, cell motility, and angiogenesis [58]. A favorable *in vivo* effect on regenerative activity has been shown in the mouse [59], rat [60], and dog [61]. These effects were seen not only after partial hepatectomy, but also in resting, healthy liver, as well. In other words regeneration occurred with HGF as the sole stimulus, whereas most other studies of liver growth factors only show an improvement in regeneration after a stimulus such as hepatic injury or partial hepatectomy.

The effects of HGF *in vivo* in the setting of cirrhosis require further study [62]. Moreover, its action is not specific to the liver; similar effects have also been demonstrated in the lung, skin, kidney, and stomach [62]. High serum levels of HGF are seen in patients with cirrhosis or fulminant hepatic failure [63], and it is possible that the cirrhotic liver is relatively unresponsive to HGF.

### Portal Venous Embolization

Because of the dual blood supply to the liver (portal vein and hepatic artery), ligation of one blood supply is possible with less risk of infarction than in tissues with blood supply from a single vessel. In a rat model that dates back to 1920, ligation of one main portal venous branch led to atrophy of the lobes supplied by the branch. Of note, the other, non-ligated lobes were stimulated to hypertrophy [64]. Since then, the importance of hepatotrophic factors found in portal blood has been well established [65].

This intervention has particular relevance to the treatment of hepatic malignancies, which, as discussed, commonly coexist with cirrhosis. Portal Branch Ligation (PBL) could cause atrophy of the affected lobe containing tumor and at the same time, prime the remaining liver to regenerate, thereby facilitating resection and improving cure rates. A further advantage is the limitation of portal tumor emboli which may occur at the time of surgery [66].

The usefulness of PBL as a preoperative intervention has been demonstrated in studies from Japan, where the incidence of HCC is high. The procedure of Portal Venous Embolization (PVE) involves injecting embolic material into one or more portal vein branches *via* a percutaneously guided catheter. Once experience is gained, this procedure can be done with relative safety [67]. One study has shown an improvement in survival and RES function in non-cirrhotic rats [68]; the same has not been shown in an animal model of cirrhosis.

Early clinical experience with this treatment modality in cirrhotic patients have been promising with significant hypertrophy of remaining lobes, and good anticancer effect [69].

### Postoperative Improvement in Regeneration

*Transforming Growth Factor Alpha (TGF -alpha)* is a cytokine involved in hepatic regeneration. Kokudo *et al.*, studied the effect of TGF-alpha on liver regeneration after hepatectomy in $CCl_4$ induced cirrhosis in the rat [70]. Regeneration in cirrhotic rats as measured by $^{3}$[H]-Thymidine incorporation was markedly impaired after hepatectomy. Cirrhotic rats treated with TGF-alpha, however, achieved regeneration comparable to that in non-cirrhotic rats. TGF-alpha had no effect on regeneration in non-cirrhotic liver. This study suggests that there is a relative deficiency of TGF-alpha in cirrhotic liver; whether this is due to decreased production, receptor downregulation, or the presence of competitive inhibitors will require further investigation.

*Putrescine* is a polyamine not only involved in hepatic regeneration, but also the growth of many tissues. Putrescine and the other polyamines appear to be essential for liver regeneration to take place [71]. Furthermore, the addition of exogenous putrescine was shown to enhance regeneration in models of acute liver disease [72]. Thus, a study was undertaken to determine the effect of putrescine at doses of 1, 10, and 100 mg/kg given before and after partial hepatectomy in a rat model of cirrhosis. Nonetheless, no effect on regeneration was seen as measured by DNA synthesis or ODC activity. Part of this lack of effect could be due to the lack of change in hepatocyte putrescine levels achieved [73].

Unfortunately, excessive regenerative activity could actually contribute to impaired liver function and lead to liver failure in the post operative period. This was suggested by Ito [74] who examined the relative expression of housekeeping and regeneration genes in a rat model following 70% hepatectomy. The housekeeping genes studied, including albumin and ornithine transcarbamylase (OTC), were significantly depressed at 24 hours after hepatectomy, at a time when accelerated DNA synthesis was beginning. This corresponded to a peak in the transcription of the oncogenes c-fos, c-myc and p53. The authors speculate that regenerating hepatocytes undergo dedifferentiation and as a result, cannot participate in liver synthetic functions. This is proposed as a putative cause for postoperative liver failure after resection. Thus augmenting regeneration may not actually be as beneficial in practice as it is in theory. Further studies examining dedifferentiation and the optimal timing to induce regeneration are required.

### SUMMARY

Liver resection in the setting of cirrhosis is not an uncommon situation, especially in the treatment of hepatocellular carcinoma, and the coexistence of cirrhosis seriously limits curability. Morbidity and mortality rates are unacceptably high in cirrhotic patients and a number of factors are likely responsible, including marginal hepatic function, portal hypertension, and impaired regeneration. Severe complications contributing to the high mortality include post operative liver failure, sepsis, and bleeding. Many studies over the last 10 years have

TABLE I Morbidity and mortality associated with liver resection in cirrhosis: several large reports

| Author (year) | Patients | Complications Liver failure* | Bleeding* | Sepsis* | Complications* | Mortality* |
|---|---|---|---|---|---|---|
| Doberneck (1982)[1] | cirrhosis various surgeries ($n=102$) | 43 (42.2) | 9 (8.8) | 19 (18.6) | 48 (47.1) | 20 (19.6) |
| Yanaga (1985)[2] | cirrhosis ($n=103$) | 13 (12.6) | NR | 16 (16.8) | 29 (28.2) | 11 (10.6) |
| Nagasue (1985)[3] | cirrhosis and HCC ($n=101$) | 10 (9.8) | 10 (9.8) | 4 (4.2) | 32 (31.7) | 9 (8.9) |
| Bismuth (1986)[4] | cirrhosis and HCC ($n=35$) | 15 (42.9) | NR | NR | 20 (47.2) | 5 (14.3) |
| Gozetti (1987)[5] | cirrhosis ($n=25$) | 3 (12.0) | 1 (4.0) | NR | NR | 4 (16.0) |
| Fan (1995)[6] | cirrhosis, major resections ($n=54$) | NR | 4 (7.4) | 18 (33.0) | 27 (50.0) | 7 (13.0) |

* Numbers in parentheses represent percentages.

NR = Not Reported.

[1] *Am. J. Surg.*, **146**, 306–309.

[2] *Ann. Surg.*, **203**(2), 148–152.

[3] *Surgery*, **99**(6), 694–701.

[4] *World J. Surgery*, **10**, 311–317.

[5] *Int. Surg.*, **72**, 82–86.

[6] *Arch. Surg.*, **130**, 198–202.

examined means of improving outcome after hepatic resection in cirrhosis. Preoperative assessment of liver function and determination of the extent of resection that can be tolerated have been studied and as a result, much has been learned. Intraoperative ultrasound and subsegmental resection appear to be beneficial surgical techniques, while the technique of hepatic inflow/outflow occlusion is being investigated for its applicability in the setting of cirrhosis. Portal venous embolization, which aids in promoting atrophy of the diseased lobe and regeneration of the remaining liver could in principle be particularly helpful in the preparation of cirrhotic patients undergoing hepatic resections. Basic research is also underway, searching for ways of improving liver function preoperatively as well as augmenting regeneration postoperatively-a number of candidates have been identified but few have been subjected to the rigors of clinical trials. While a number of advancements have allowed a slight increase in survival, many approaches remain to be explored in an attempt to reduce the high morbidity and mortality of liver resection in cirrhosis and increase the cure rate of hepatic malignancies.

### *References*

[1] MacIntosh, E. M. and Minuk, G. Y. (1992). Hepatic resection in patients with cirrhosis and Hepatocellular carcinoma. *Surg. Gynecol, Obst.*, **174**, 245–254.

[2] Gozzeti, G. and Mazziotti, A. (1987). Hepatic resection for tumors in cirrhotic livers. *Int. Surg.*, **72**, 82–86.

[3] Bismuth, H., Houssin, D. *et al.* (1986). Liver resection if cirrhotic patients: A Western experience. *World J. Surg.*, **10**(2), 311–317.

[4] Fujio, N., Sakai, K. *et al.* (1989). Results of Treatment of patients with hepatocellular carcinoma with severe cirrhosis of the liver. *World J. Surg.*, **13**, 211–218.

[5] Yamanaka, N., Okamoto, E. *et al.* (1994). A Prediction scoring system to select the surgical treatment of liver cancer: Further refinement based on ten years of use. *Ann. Surg.*, **219**(4), 342–346.

[6] Kinami, Y. and Takashima, S. (1986). Hepatic resection for hepatocellular carcinoma associated with liver cirrhosis. *World J. Surg.*, **10**, 294–301.

[7] Cameron, G. R. (1936). $CCl_4$ cirrhosis in relation to liver regeneration. *J. Pathol.*, **42**, 1–21.

[8] Nagasue, N., Yukaya, H. *et al.* (1987). Human liver regeneration after major hepatic resection: A study of normal livers and livers with chronic hepatitis and cirrhosis. *Ann. Surg.*, **206**, 30–39.

[9] Minuk, G. Y. (1993). Gamma-Aminobutyric Acid and the Liver. *Dig. Dis.*, **11**, 45–54.

[10] Zieve, L., Shekelton, M. *et al.* (1985). Ammonia, Octanoate, and a Mercaptan depress regeneration of normal rat liver after partial hepatectomy. *Hepatology*, **5**(1), 28–31.

[11] Aranha, G. V., Sontag, S. J. and Greenlee, H. B. (1982). Cholecystectomy in cirrhotic patients: A formidable operation. *Am. J. Surg.*, **143**, 55–60.

[12] Shwartz, S. I. (1981). Biliary tract surgery and cirrhosis: A critical combination. *Surgery*, **90**(4), 577–579.

[13] Kanematsu, T., Takenoka, K. *et al.* (1985). Acute portal hypertension associated with liver resection. *Arch. Surg.*, **120**, 1303–1305.

[14] Tobe, T. (1986). Hepatectomy in patients with cirrhotic livers: clinical and basic observations. *Surg. Annu.*, **121**, 515–521.

[15] Ozawa, K., Yamaoka, Y. *et al.* (1973). Clinical application of cytochrome a ($+a_3$) assay of mitochondria from liver specimens: An aid in determining metabolic tolerance of liver remnant for hepatic resection. *Ann. Surg.*, **180**(6), 868–876.

[16] Stimson, R. E. J., Pellegrini, C. A. and Way, L. W. (1987). Factors affecting the morbidity of elective liver resection. *Am. J. Surg.*, **153**, 189–194.

[17] Yamanaka, N., Okamoto, E. *et al.*, A Multiple regression equation for prediction of post-hepatectomy liver failure. *Ann. Surg.*, **200**(5), 658–663. (Nov. 1984).

[18] Yamanaka, N., Okamoto, E. *et al.* (1994). A prediction scoring system to select the surgical treatment of liver cancer: Further refinement based on ten years of use. *Ann. Surg.*, **219**(4), 342–346.

[19] Doberneck, R. C., Sterling, W. A. and Allison, D. C. (1983). Morbidity and mortality after operation in nonbleeding cirrhotic patients. *Am. J. Surg.*, **146**, 306–309.

[20] Yanaga, K., Kanematsu, T. *et al.* (1986). Intraperitoneal septic complications after hepatectomy. *Ann. Surg.*, **203**(2), 148–152.

[21] Nagasue, N., Yukaya, H., Ogawa, Y. *et al.* (1986). Clinical experience with 118 hepatic resections for hepatocellular carcinoma. *Surgery*, **99**(6), 694–701.

[22] Fan, S., Lai, E. C. S. *et al.* (1995). Hospital mortality of major hepatectomy for hepatocellular carcinoma associated with cirrhosis. *Arch. Surg.*, **130**, 198–202.

[23] Sirinek, K. R., Martin, E. W. and Thomford, N. R. (1976). Peripheral Vasopressin for safe and adequate control of portal hypertension during shunt operations. *Am. J. Surg.*, **131**, 103–108.

[24] Scudamore, C. H., Randall, T. E. *et al.* (1995). Aprotinin reduces the need for blood products during liver transplantation. *Am. J. Surg.*, **169**, 546–549.

[25] Belghiti, J., DiCarlo, I. *et al.* (1994). A Ten year experience with hepatic resection in 338 patients: evolution in indications and of operative mortality. *Eur. J. Surg.*, **160**, 277–282.

[26] Sitzmann, J. V. and Greene, P. S. (1994). Perioperative predictors of morbidity following hepatic resection for neoplasm. *Ann. Surg.*, **219**(1), 13–17.

[27] McIntyre, N. (1983). The limitations of conventional liver function tests. *Sem. Liver. Dis.*, **3**(4), 265–274.

[28] Bircher, J. (1983). Quantitative assessment of deranged hepatic function: A missed opportunity? *Sem. Liver Dis.*, **3**(4), 275–284.

[29] Hemming, A. W. and Scudamore, C. H. (1992). Indocyanine Green clearance as a predictor of successful hepatic resection in cirrhotic patients. *Am. J. Surg.*, **163**, 515–518.

[30] Mimura, H., Takakura, N. *et al.* (1986). Determination of the extent of feasible hepatic resection from hepatic blood flow. *World J. Surg.*, **10**, 302–310.

[31] Okamoto, E., Kyo, A. *et al.* (1983). Prediction of the safe limits of hepatectomy by combined volumetric and functional measurements in patients with impaired hepatic function. *Surgery*, **95**(5), 586–591.

[32] Makuuchi, M., Hasegawa, H. and Yamazaki, S. (1985). Ultrasonically guided subsegmentectomy. *Surg. Gynecol. Obst.*, **98**, 942–948.

[33] Parker, G. A. and Lawrence, W. Jr. (1989). Intraoperative ultrasound of the liver affects operative decision making. *Ann. Surg.*, **209**(5), S69–77.

[34] Solomon, M. J., Steven, M. S. and Godinger, S. (1994). Does intraoperative ultrasound change surgical decision making during liver resection? *Am. J. Surg.*, **168**, 307–310.

[35] Isozaki, H. and Okajinik (1995). Experimental study of liver injury after partial hepatectomy with intermittent or continuous hepatic vascular occlusion. Differences in tolerance to ischemia between normal and cirrhotic livers. *Eur. Surg. Res.*, **27**(5), 313–322.

[36] Nagasue, N., Yukawa, H. *et al.* (1984). Tolerance of the cirrhotic liver to normothermic ischemia. A clinical study of 15 patients. *Am. J. Surg.*, **147**, 772–775.

[37] Nagasue, N., Yakaya, H., Ogawa, Y. and Okita, M. (1985). Segmental and subsegmental resection of the cirrhotic liver under hepatic inflow and outflow occlusion. *Br. J. Surg.*, **72**(1), 565–568.

[38] Izumi, R., Yabushita, K., Shimizu, K. *et al.* (1993). Hepatic resection using a water jet dissector. *Surg. Today*, **23**(1), 31–35.

[39] Rau, H. G., Schardey, H. M. and Schildberg, F. (1995). A comparison of different techniques for liver resection: blunt dissection, ultrasonic aspirator, and jet-cutter. *Eur. J. Surg. Oncol.*, **21**(2), 183–187.

[40] Lin, T. Y. (1974). Results in 107 hepatic lobectomies with a preliminary report on the use of a clamp to reduce blood loss. *Ann. Surg.*, **177**(4), 413–421.

[41] Shimada, M., Matsumada, T. and Takematami, A. (1987). A new approach for liver surgery: Transdiaphragmantic hepatectomy for cirrhosis patients with HCC. *Arch. Surg.*, **130**, 157–159.

[42] Callea, F., Brisigotti, M. *et al.* (1991). Cirrhosis of the liver: A regenerative process. *Dig. Dis. Sci.*, **36**(9), 1287–1293.

[43] MacIntosh, E. L., Gauthier, T. and Minuk, G. Y., Hepatic fibrosis as a predictor of hepatic regenearation activity after partial hepatectomy in the rat. *Hepatology*, **17**(2), 307–310.

[44] Wu, J. and Danielsson, A. (1994). Inhibition of hepatic fibrogenesis: A review of pharmacologic candidates. *Scand. J. Gastroent.*, **29**, 385–391.

[45] Tanner, M. S., Jackson, D. and Mowat, A. P. (1981). Hepatic collagen synthesis in a rat model of cirrhosis and its modification by Colchicine. *J. Pathol.*, **135**, 179–187.

[46] Rojkind, M. and Kushenobick (1975). Effect of Colchicine on collagen, albumin and Transferrin synthesis by cirrhotic rat liver. *Biochim. Biophys. Acta*, **378**, 415–23.

[47] Kershenobich, D., Vargas, F. *et al.* (1988). Colchicine in the treatment of cirrhosis of the liver. *N. Eng. J. Med.*, **318**(26), 1709–1713.

[48] Peterson, T. C., Pentoxyfilline prevents fibrosis in an animal model and inhibits platelet-derived growth factor-driven proliferation of fibroblasts. *Hepatology*, 1993 March, **17**(3), 486–493.

[49] Peterson, T. C. and Isbrucker, R. A., *In vitro* effect of platelet-derived growth factor on fibroproliferation and effect of cytokine antagonists. *Immunopharmacology*, 1994 Nov–Dec, **28**(3), 259–70.

[50] Hernandez, R. and Diasz-Munoz, H. (1991). Adenosine partially prevents cirrhosis by $CCl_4$ in rats. *Hepatology*, **12**, 242–248.

[51] Ferenci, P., Minuk, G. Y. *et al.* (1984). Identification of an acceptor system for GABA on isolated rat hepatocytes. *Hepatology*, **4**(2), 180–185.

[52] Minuk, G. Y. and Gauthier, T. (1993). The effect of GABA on hepatic regenerative activity following partial hepatectomy in rats. *Gastroenterology*, **104**, 217–221.

[53] Minuk, G. Y. and Sarjeant, E. J. (1988). The effect of (a) Neomycin and Lactulose treatments on systemic and portal serum GABA levels in rats and (b) pH changes on $^{3}$[H]-GABA binding to isolated rat hepatocytes. *Clin. Invest. Med.*, **95**, 1356–1363.

[54] Minuk, G. Y., Gauthier, T. *et al.*, Ciprofloxacin prevents the inhibitory effects of acute ethanol exposure on hepatic regeneration in the rat. *Hepatology*, **22**(6), 1797–1800, (1995 Dec.).

[55] MacIntosh, E. L., Gauthier, T. and Minuk, G. Y. (1992). Selective Bowel decontamination does not alter hepatic regeneration in rats. *Gastroenterology*, **102**, 1403–1405.

[56] Minuk, G. Y. and Zhang, M. N. (1996). Effect of Hepatic Stimulator Substance, Herbal Medicine, Selenium/Vitamin E, and Ciprofloxacin on cirrhosis in the rat. *Gastroenterology*, **110**, 1150–1155.

[57] Ishii, T., Sato, M. *et al.* (1995). Hepatocyte Growth Factor stimulates liver regeneration and elevates blood protein level in normal and partially hepatectomized rats. *J. Biochem.*, **117**, 1105–1112.

[58] Hoffman, A. L., Rosen, H. R. *et al.* (1994). Hepatic Regeneration: Current Concepts and clinical implications. Seminars in liver disease. **14**(2), 190–212.

[59] Roos, F., Ryan, A. M. *et al.* (1995). Induction of liver growth in normal mice by infusion of hepatocyte growth factor/scatter factor. *Am. J. Physiol.*, **268**, G380–386.

[60] Fujiwara, K., Nagoshi, S. *et al.* (1993). Stimulation of liver growth by exogenous human HGF in normal and partially hepatectomized rats. *Hepatology*, **18**(6), 1443–1449.

[61] Kobayashi, Y., Hamanoue, M. *et al.* (1996). Induction of Hepatocyte growth by intraportal infusion of HGF into beagle dogs. *Biochem. Biophys. Res. Comm.*, **220**, 7–12.

[62] Miller, C. M. and Boros, P. (1995). Hepatocyte growth factor: A multifunctional cytokine. *Lancet*, **345**, 293–295.

[63] Fausto, N., Laird, A. D. *et al.* (1995). Role of growth factors and cytokines in hepatic regeneration. *FASEB J.*, **9**, 1527–1535.

[64] Rous, P. and Larimore, L. D. (1920). Relation of portal blood to liver maintenance. A demonstration of liver atrophy conditional on compensation. *J. Exp. Med.*, **31**, 609–632.

[65] Rozga, J., Jeppsson, B. and Bengmark, S. (1987). Portal branch ligation in the rat: Reevaluation of a model. *Am. J. Path.*, **125**(2), 300–308.

[66] Kinoshita, H., Sakai, K., Hirohashi, K. *et al.* (1986). Preoperative Portal vein embolization for hepatocellular carcinoma. *World J. Surg.*, **10**, 803–808.

[67] Lee, K. C., Kinoshtia, H., Hirohashi *et al.* (1993). Extension of surgical indications for hepatocellular carcinoma by portal vein embolization. *World. J. Surg.*, **17**(1), 109–115.

[68] Tanaka, H. and Kinoshita, H. (1994). Increased safety by two-stage hepatectomy with preoperative portal vein embolization in rats. *J. Surg. Research*, **57**, 687–692.

[69] Kinoshita, H., Sakai, K., Hirohashi, K. *et al.* (1986). Preoperative Portal vein embolization for hepatocellular carcinoma. *World J. Surg.*, **10**, 803–808.

[70] Kokudo, N., Kothary, P. C. *et al.* (1992). Transforming Growth Factor improves hepatic DNA synthesis after hepatectomy in cirrhotic rats. *J. Surg. Res.*, **52**, 648–655.

[71] Luk, G. D. (1986). Essential role of polyamine metabolism in hepatic regeneration. *Gastroenterology*, **90**, 1261–1267.

[72] Diehl, A. M., Abdo, S. and Bram, N. (1990). Supplemental Putrescine reverses ethanol-associated inhibition of liver regeneration. *Hepatology*, **12**, 633–637.

[73] MacIntosh, E. L., Gauthier, T. and Minuk, G. Y. (1992). Liver regeneration and effect of exogenous Putrescine on regenerative activity after partial hepatectomy in cirrhotic rats. *Hepatology*, **16**(6), 1428–33.

[74] Ito, Y., Hayashi, H. *et al.* (1991). Depression of liver specific gene expression in regenerating rat liver: A putative cause for liver dysfunction after hepatectomy. *J. Surg. Res.*, **51**, 143–147.

